# Emergence of ST1076 as a dominant high-risk clone carrying *bla*_*KPC*–2_ in carbapenem-resistant *Pseudomonas aeruginosa* from Deqing, Zhejiang, China: a 5-year genomic epidemiology study

**DOI:** 10.3389/fmicb.2026.1839819

**Published:** 2026-07-09

**Authors:** Xiaolan Shou, Xiangchen Li, Hongyin Yang, Xiaofan Zhang

**Affiliations:** 1 Department of Clinical Laboratory, Deqing People’s Hospital (Deqing Campus, Sir Run Run Shaw Hospital, School of Medicine, Zhejiang University), Deqing, Zhejiang, China; 2 Jiaxing Key Laboratory of Clinical Laboratory Diagnostics and Translational Research, Affiliated Hospital of Jiaxing University, Jiaxing, China; 3 Department of Clinical Laboratory, Feicheng Hospital of Traditional Chinese Medicine, Taian, Shandong, China

**Keywords:** carbapenem-resistant *Pseudomonas aeruginosa*, ceftazidime/avibactam, KPC-2, molecular epidemiology, nosocomial transmission, ST1076

## Abstract

**Background:**

Carbapenem-resistant *Pseudomonas aeruginosa* (CRPA) is a major healthcare-associated pathogen with high multidrug resistance and mortality. Regional clonal spread of high-risk lineages carrying carbapenemase genes poses significant challenges for infection control.

**Methods:**

We analyzed 100 non-duplicate CRPA clinical isolates collected from June 2020 to December 2024 in Deqing, Zhejiang Province, China. Antimicrobial susceptibility was determined by automated systems, and molecular characterization was performed via whole-genome sequencing, multilocus sequence typing (MLST), core-genome single nucleotide polymorphism (SNP) phylogeny, resistance and virulence gene profiling, and phylogeographic analysis.

**Results:**

The observed resistance rates were 95% for imipenem and 91% for meropenem. Carbapenemase-producing isolates comprised 44% (44/100), with *bla*_*KPC*–2_ predominating (95.5%, 42/44). MLST identified 44 sequence types, including novel ST5216. ST1076 was the dominant clone (23%, 23/100), followed by ST463 (17%). Among ST1076 isolates, 87.0% carried *bla*_*KPC*–2_. Core-genome SNP analysis revealed tight clustering of 22/23 ST1076 isolates (4–105 SNPs), suggesting possible prolonged nosocomial transmission centered in the Intensive Care Unit (ICU) since mid-2020. Phylogeographic analysis showed strong clustering with ST1076 strains from Ningbo and Hangzhou, supporting intra-provincial dissemination within Zhejiang. Virulence profiling demonstrated high prevalence of T3SS effectors (*exoT* 100%, *exoY* 97%) and frequent *oprD* inactivation.

**Conclusion:**

This study reveals the emergence and sustained ICU-associated transmission of *bla*_*KPC*–2_-harboring ST1076 CRPA as a regionally dominant clone in Zhejiang, China, while preserving high susceptibility to ceftazidime/avibactam (CZA). Enhanced local surveillance and strict infection control measures are urgently needed to prevent further spread of this high-risk lineage.

## 1 Introduction

*P. aeruginosa* is an important opportunistic pathogen with widespread distribution in nature and has the potential to cause community-acquired infections; however, severe infections caused by *P. aeruginosa* are primarily hospital-acquired (Lister et al., 2009). Given that high rates of antibiotic resistance are frequently observed in pathogen samples from hospital-acquired infections. Therefore, selecting the appropriate antibiotic is crucial for treating *P. aeruginosa* infections. Carbapenems antibiotics were considered to be one of the last lines for *P. aeruginosa* treatment (Crandon et al., 2016). However, *P. aeruginosa* possesses the ability to acquire resistance genes horizontally, developing resistance to carbapenems. Thus, the emergence of CRPA poses a significant public health challenge worldwide (Reyes et al., 2023).

Previous reports have shown that CRPA strains possess multiple mechanisms of resistance to carbapenem antibiotics: first, overexpression of the MexAB-OprM efflux pump leads to drug efflux (Livermore, 2002); second, overproduction of AmpC combined with the inactivation of *OprD* (Tenover et al., 2022); third, the production of carbapenemases is the most prevalent resistance mechanism, as it significantly alters the efficacy of commonly used anti-Pseudomonas drugs (including ceftazidime, cefepime, etc.) and novel β-lactam/β-lactamase inhibitor combinations (ceftazidime/avibactam, etc.) (Li et al., 2022; Li et al., 2023; Zhou L. et al., 2025). Metallo-β-lactamases (MBLs) are a common type of carbapenemases in CRPA (Bush, 2013). Notably, imipenemase (IMP) are carried by mobile genetic elements, which also harbor resistance genes against other antibiotic classes (Escandón-Vargas et al., 2017). In addition, the detection rate of Class A carbapenemases (particularly *Klebsiella pneumoniae* carbapenemase, or KPC) in CRPA strains has been gradually increasing (Zhou et al., 2024). The coexistence of KPC and MBL in *P. aeruginosa* confers resistance to most β-lactam antibiotics (including novel β-lactam/β-lactamase inhibitors such as CZA), although aztreonam-avibactam (particularly against *bla*_*KPC*_- and *bla*_*VIM*_-producing strains) and cefiderocol may remain viable options, these regimens present significant clinical challenges (Blanco-Martín et al., 2024; Liu et al., 2025).

Sequence types (STs) are widely used for genotyping *P. aeruginosa* and tracking the global dissemination of high-risk clones. Notably, several high-risk CRPA clones (such as ST235 and ST463) are associated with significantly higher clinical death rates (Recio et al., 2018; Recio et al., 2021). The ST463 is the most common clonal type among CRPA isolates in China and often carries *bla*_*KPC*–2_ or MBLs (Reyes et al., 2023). However, research on ST1076 CRPA remains limited, although this clone has been identified as an emerging high-risk carbapenemase-carrying clone. ST1076 has been reported to carry *bla*_*KPC*–2_ and *bla*_*KPC*–3_, which are located on a transferable IncP-2 megaplasmid (Chen et al., 2025). Moreover, ST1076 has become the predominant clone in certain regions, accounting for 29.3% of CRPA strains in Ningbo, China (Qiao et al., 2025). Additionally, a previous report described an outbreak of ST1076-PA in a neonatal ICU in Switzerland; the isolate carried *bla*_*KPC*–2_, a carbapenemase that, particularly when coexisting with MBLs, confers resistance to the vast majority of β-lactam antibiotics (Cameron et al., 2022; Liu et al., 2025).

Despite the global emergence of CRPA strains, the molecular epidemiology and resistance mechanisms of regional isolates particularly those belonging to the ST1076 clone remain poorly understood in Deqing City, Zhejiang Province. Furthermore, the phylogenetic relationships between local ST1076 isolates and those from other regions within Zhejiang Province have not been characterized. Thus, this study aims to elucidate the molecular epidemiological features and resistance mechanisms of CRPA isolates from this region, and to delineate the phylogenetic context of ST1076 CRPA strains in relation to regional isolates. The findings are expected to provide new insights into the transmission dynamics of CRPA and infection control measures to prevent the clinical spread of ST1076 CRPA.

## 2 Materials and methods

### 2.1 Bacterial isolates

Between June 2020 and December 2024, 100 non-duplicate clinical CRPA isolates were collected from various specimens at a hospital in Deqing, China. Non-duplicate CRPA strains were defined as one CRPA strain carried by each patient during a single hospitalization. CRPA isolates defined as resistant to either meropenem or imipenem according to the Clinical and Laboratory Standards Institute (CLSI) breakpoints (Clinical and Laboratory Standards Institute [CLSI], 2026). All strains were identified as *P. aeruginosa* using matrix-Assisted laser desorption/ionization time-of-flight mass spectrometr apparatus (MALDI-TOF MS; Zybio (Chongqing) Biotechnology Co., Ltd., China).

### 2.2 Antimicrobial susceptibility testing

Antimicrobial susceptibility testing and species confirmation were performed using the VITEK 2 system (VITEK2; bioMerieux, France). The VITEK 2 system was selected because it is the standard automated platform we use in our clinical laboratory for routine antimicrobial susceptibility testing, and all data were collected retrospectively from routine clinical records. The minimum inhibitory concentration (MIC) of piperacillin-tazobactam, ticarcillin-clavulanate, ceftazidime, cefepime, imipenem, meropenem, ciprofloxacin, levofloxacin, tobramycin, and amikacin were determined using VITEK2 (bioMerieux) according to CLSI guidelines (Clinical and Laboratory Standards Institute [CLSI], 2026). *P. aeruginosa* ATCC 27853 was used as quality control. The multidrug-resistant (MDR), extensively drug-resistant (XDR) and pandrug-resistant (PDR) phenotypes were defined according to the criteria established by Magiorakos et al. (2012).

### 2.3 Whole-genome sequencing (WGS) and genome assembly

The genomic DNA of all CRPA isolates was extracted using QIAamp DNA Mini Kit (Qiagen, Hilden, Germany). Sequencing was performed on the Illumina NovaSeq 6000 (San Diego, California, United States). After quality control of the raw data using fastp v0.23.2 (Chen, 2025), genome assembly was completed with Unicycler v0.5.1 under default parameters (Wick et al., 2017). The assembly quality was evaluated by QUAST v5.0.2 (Gurevich et al., 2013), CheckM v1.1.3 and fastANI v1.34, with *P. aeruginosa* PAO1 (NCBI RefSeq assembly: GCF_000006765.1) as the reference genome.

### 2.4 Bioinformatics and phylogenetic analysis

Multilocus sequence typing (MLST) was performed using mlst v2.19.0^1^ with the PubMLST database scheme (Jolley et al., 2018). Antimicrobial resistance genes were screened using ABRicate v1.0.0^2^ against the Comprehensive Antibiotic Resistance Database (CARD) (Alcock et al., 2023). Prokka v1.11 was used to annotate the location and function of the genes (Seemann, 2014). Screening virulence factors (VFs) using the Virulence Factors Database (VFDB) (Zhou S. et al., 2025). The genome sequences of the strains were uploaded to the BV-BRC (Bacterial and Viral Bioinformatics Resource Center) database, and source tracking analysis was performed using the Similar Genome Finder tool provided by the platform (Olson et al., 2023).

To determine the genetic location of *bla*_*KPC*_, putative plasmid sequences were reconstructed from Illumina short-read assemblies using MOB-suite v3.19 (mob-recon) with default parameters. Contigs identified as plasmid-derived were classified by relaxase and replicon typing; resistance genes on these contigs were annotated with ABRicate v1.0.0 against the CARD database. Isolates in which *bla*_*KPC*_ was detected on a chromosomal contig were recorded as chromosomal carriers. Conjugative transfer (*tra*) and mating pair formation (*mpf*) gene clusters were assessed to infer mobility.

Core genome alignment and single nucleotide polymorphism (SNP) calling were performed with Parsnp v1.2 (HarvestTools suite), using *P. aeruginosa* PAO1 (NCBI RefSeq assembly: GCF_000006765.1) as the reference genome. Putative recombination regions were removed with Gubbins v2.1.0. A maximum-likelihood phylogenetic tree was subsequently constructed from the resulting core SNP alignment using RAxML v8.2.9 (Stamatakis, 2014), under the GTR+G substitution model with 1,000 bootstrap replicates. The final tree was visualized and annotated using the Interactive Tree of Life (iTOL) online platform (Letunic and Bork, 2024). cgSNP distances were calculated from the recombination-filtered alignment (Treangen et al., 2014). A cgSNP threshold of ≤ 25 was applied to define closely related transmissions, consistent with thresholds commonly used in *P. aeruginosa* nosocomial outbreak investigations and patient-to-patient transmission studies (Romano-Bertrand et al., 2025; Graña-Miraglia et al., 2026). Furthermore, a molecular network was constructed by PopArt software based on the Median-joining algorithm (Leigh and Bryant, 2015).

For Bayesian time-scaled phylogenetic inference, a time-scaled phylogenetic tree was reconstructed using BactDating v1.0.12 under the mixed additive uncorrelated relaxed clock (mixedcarc) model with a coalescent prior (Didelot et al., 2018). This model was selected because it is recommended for bacterial phylogenies with heterogeneous substitution rates across lineages and has been widely applied in recent studies on *P. aeruginosa* and related pathogens. Prior to analysis, root-to-tip regression was performed in BactDating to confirm a strong temporal signal. MCMC chains were run for 108 iterations to ensure sufficient sampling, achieving convergence for all estimated parameters with effective sample sizes (ESS) > 200 and good chain mixing.

### 2.5 Accession numbers

The datasets presented in this study can be found in online repositories. Genome sequences of all strains have been deposited in the NCBI database under BioProject accession numbers PRJNA1434323.

## 3 Results

### 3.1 Overview of CRPA isolates

In this study, 100 non-duplicate CRPA clinical isolates collected from June 2020 to December 2024 were included. The isolates were predominantly recovered from respiratory specimens (*n*81, 81.0%), followed by urine (*n*7, 7.0%), bronchoalveolar lavage fluid (BALF) (*n*4, 4.0%), wound secretions (*n*4, 4.0%), blood (*n*2, 2.0%), feces (*n*1, 1.0%), and bile (*n*1, 1.0%).

Antimicrobial susceptibility testing results show that the resistance rate for imipenem is 95% (95/100), while the resistance rate for meropenem is 91% (91/100). Resistance rates to other agents were as follows: piperacillin-tazobactam 64% (64/100), ticarcillin-clavulanate 80% (80/100), ceftazidime 42% (42/100), ceftazidime-avibactam 34% (34/100), cefepime 49% (49/100), ciprofloxacin 45% (45/100), levofloxacin 58% (58/100), tobramycin 17% (17/100) and amikacin 6% (6/100) (Table 1). Overall, while carbapenem resistance was nearly universal (imipenem 95%, meropenem 91%), a substantial proportion of isolates (e.g., 58% to ceftazidime and 94% to amikacin) remained susceptible or intermediate to non-carbapenem β-lactams (Table 1). Amikacin retained high *in vitro* activity against the majority of strains. Among the 100 CRPA isolates, 69% (69/100) of the isolates were classified as multidrug-resistant (MDR), 10% (10/100) as extensively drug-resistant (XDR), and 1% (1/100) as pandrug-resistant (PDR) (Supplementary Figure S1A).

**TABLE 1:** *In vitro* activities of antibiotics against *Pseudomonas aeruginosa* isolates in this study.

Antibiotics	R%	I%	S%
PTZ	64%	12%	24%
TZP	80%	9%	11%
CAZ	42%	20%	38%
CZA	34%	0%	66%
FEP	49%	13%	38%
IPM	95%	1%	4%
MEM	91%	6%	3%
CIP	45%	15%	40%
LEV	58%	12%	30%
TOB	17%	3%	80%
AMK	6%	3%	91%

R%, percentage of resistant isolates; I%, percentage of intermediate isolates; S%, percentage of susceptible isolates; PTZ, piperacillin-tazobactam; TZP, ticarcillin-clavulanate; CAZ, ceftazidime; CZA, ceftazidime-avibactam; FEP, cefepime; IPM, imipenem; MEM, meropenem; CIP, ciprofloxacin; LEV, levofloxacin; TOB, tobramycin; AMK, amikacin.

### 3.2 Phylogenetic structure and genomic profiling of antibiotic resistance and virulence factors in CRPA isolates

**FIGURE 1:**
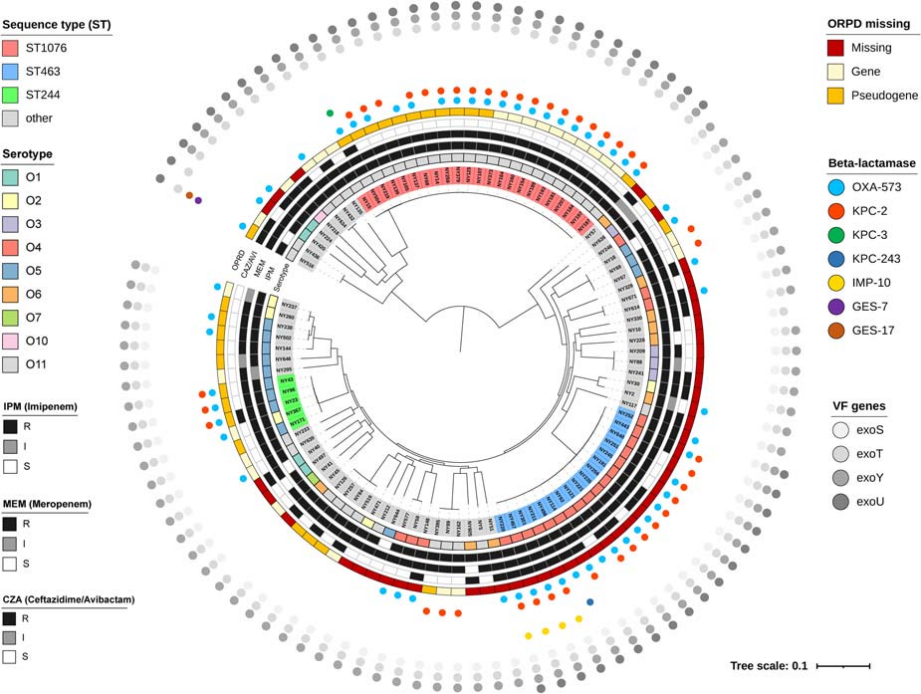
Phylogenetic overview of the collected CRPA isolates in Deqing, China. The phylogenetic tree was rooted using the midpoint method. The scale bar indicates the number of nucleotide substitutions per site. The colored backgrounds of the tree nodes represent the Sequence Types (STs). From the inner to the outer circles, colored squares indicate the Serotype, susceptibility profiles to Imipenem (IPM), Meropenem (MEM), and Ceftazidime/Avibactam (CZA), as well as the detection result of the OPRD gene. Furthermore, colored circles denote the presence of different types of beta-lactamases and exo virulence genes.

Phylogenetic analysis of the 100 CRPA isolates revealed considerable clonal diversity (Figure 1). A total of 44 distinct STs were identified, including one novel ST, designated ST5216 (GenBank accession: JBLOGV000000000.1). Among these, seven STs comprised more than three isolates each. ST1076 was the predominant clone (23%, 23/100), followed by ST463 (17%, 17/100).

Carbapenemase genes were identified in 44 out of 100 CRPA genomes, all of which were KPC variants (Figure 1). Among the detected variants, *bla*_*KPC*–2_ accounted for 95.5% (42/44), followed by *bla*_*KPC*–3_ (1/44, 2.2%) and *bla*_*KPC*–243_ (1/44, 2.2%). Notably, 87.0% (20/23) of ST1076 isolates harbored *bla*_*KPC*–2_ (Supplementary Figure S1B). All ST1076 strains were resistant to imipenem and meropenem, whereas 95.7% (22/23) remained susceptible to ceftazidime/avibactam (CZA). In contrast, although 82.4% (14/17) of ST463 isolates produced KPC-2, only 29.4% (5/17) showed susceptibility to CZA.

In addition, the prevalence of key virulence genes (*exoS*, *exoT*, *exoY*, and *exoU*) was 68, 100, 97, and 48%, respectively (Figure 1). Notably, *exoU*-carrying CRPA strains may be associated with serotypes O4 and O11. Furthermore, we examined the genetic integrity of the outer membrane porin gene *oprD* in all 100 CRPA isolates. While *oprD* homologs (>89% identity to PAO1) were detected in all strains, structural analysis revealed that only 26 *oprD* genes were intact, 28 were pseudogenes, and 46 harbored unannotated frameshift mutations or premature stop codons.

### 3.3 Characterization of the *bla*_*KPC*_-harboring plasmids

The *bla*_*KPC*_ gene was identified on a plasmid in 90.9% (40/44) of the CPPA isolates. The remaining four isolates (9.1%), including two each of ST1816 and ST244, harbored the gene on the chromosome. The 40 plasmid-borne *bla*_*KPC*_ genes were distributed across two closely related plasmid clusters, designated AF809 and AE788, both belonging to the IncU incompatibility group (Supplementary Table S1). Notably, no additional resistance genes were detected on any of the *bla*_*KPC*_-carrying plasmid contigs, indicating that these IncU platforms function primarily as dedicated KPC carbapenemase vectors rather than multidrug resistance accumulation elements.

Cluster AF809 (*n* = 21) was exclusively found in ST1076 isolates and harbored *bla*_*KPC*–2_ (*n* = 20) or *bla*_*KPC*–3_ (*n* = 1, strain NY504). These plasmids were substantially larger, ranging from 389,162 to 394,990 bp, with a mean GC content of 56.6%. They carried an additional rep_cluster_1115 replicon, encoded an MPF_*I*_-type mating pore, but lacked a complete conjugative transfer (*tra*) system and were classified as non-mobilizable. Cluster AE788 (*n* = 19) was detected in ST463 (*n* = 15, including 14 *bla*_*KPC*–2_ and 1 *bla*_*KPC*–243_), ST1212 (*n* = 3, all *bla*_*KPC*–2_), and ST244 (*n* = 1, *bla*_*KPC*–2_. In contrast to those in AF809, plasmids in AE788 were substantially smaller (33,008–52,929 bp) with a mean GC content of 59.0%. These plasmids harbored only the IncU replicon, lacked an identifiable MPF module, and were predicted to be non-mobilizable.

### 3.4 Molecular epidemiology analysis of the ST1076 CRPA outbreak

**FIGURE 2:**
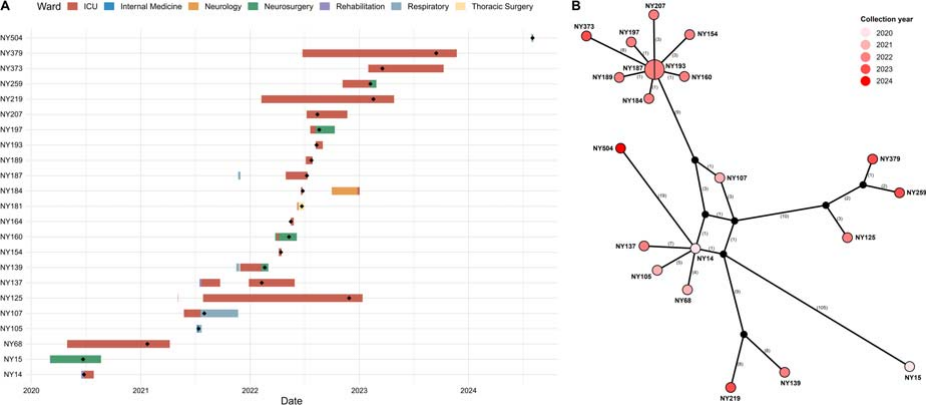
Molecular epidemiological analysis of 23 ST1076 CRPA isolates. **(A)** The ward and hospitalization timeline of the cases. Different wards are represented by distinct colors. Black diamonds indicate sampling time points. **(B)** The median-joining network, based on the cgSNP analysis, depicts 23 carbapenem-resistant Pseudomonas aeruginosa ST1076 strains. Circle size and color correspond to haplotype abundance and sampling year, respectively. Black circles represent unsampled or extinct haplotypes. Numbers in parentheses along the branches indicate the pairwise SNP distances between haplotypes.

ST1076 was identified as the dominant clone, accounting for 23% (23/100) of the CRPA isolates collected over the 2020–2024 study period. Hospitalization records of the 23 ST1076 patients revealed that 20 of them had documented ICU exposure during their admission periods (Figure 2A). Only NY105 and NY504 lacked any ICU stay.

Core-genome SNP (cgSNP) analysis showed pairwise differences ranging from 4 to 105 SNPs among the 23 ST1076 isolates (Figure 2B). Excluding the outlier NY15, 22 of the 23 isolates formed a single, tightly linked transmission chain, with pairwise distances of fewer than 25 cgSNPs spanning over 4 years. NY14, isolated on the day of ICU admission (25 June 2020) following transfer from the rehabilitation ward, was the earliest detected isolate.

Integrating the molecular network features with detailed patient hospitalization timelines, we observed that the transmission chain originated with NY14 in mid-2020 and propagated continuously through repeated ICU admissions and inter-ward transfers. Highly related isolates were subsequently recovered from patients with overlapping or sequential ICU stays, indicating sustained nosocomial dissemination centered in the ICU environment. Key bridging cases included long-stay patients such as NY68 (ICU from April 2020 to April 2021), NY107, NY125 (extended ICU stay until January 2023), NY137, NY139, NY154–NY197 (dense clustering in 2022), NY207, NY219, NY259, NY373, and NY379 (last documented case in September 2023).

### 3.5 Geographic clustering and ancestral introduction of the Deqing ST1076 clone

**FIGURE 3:**
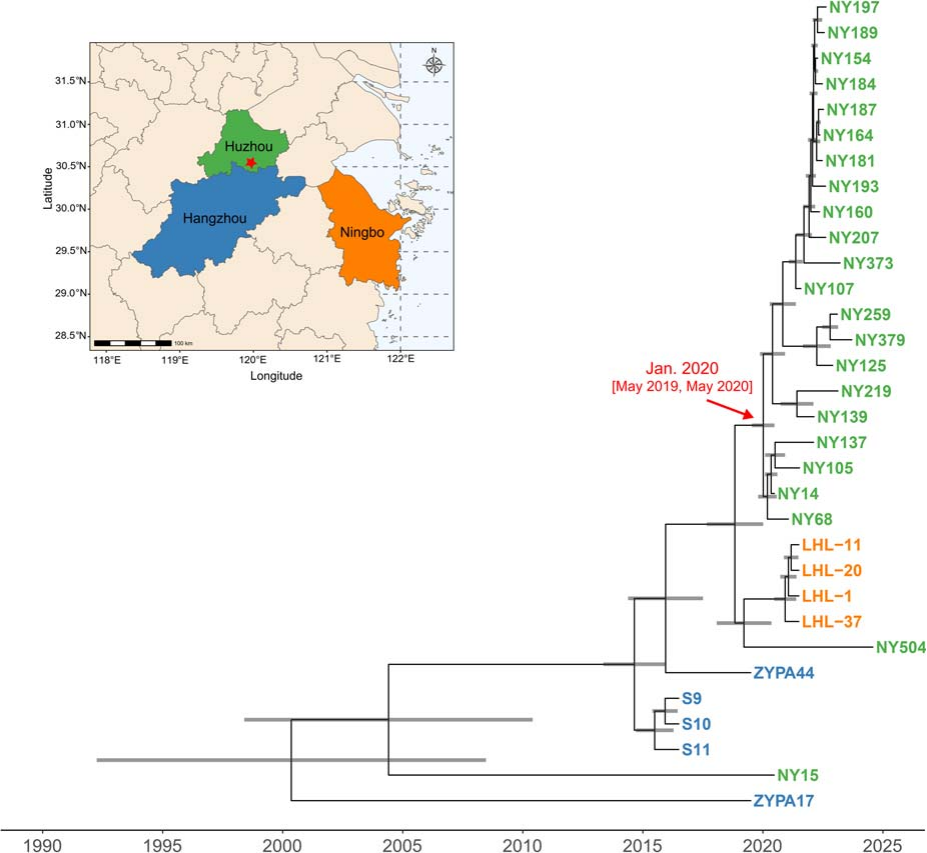
Dated phylogenetic tree of the 23 ST1076 CRPA isolates in Deqing and the 9 closely related strains isolated from Hangzhou and Ningbo city. The map on the upper left shows the location of these three areas. The color of the terminal branch node of the phylogenetic tree is consistent with the color on the map, representing the area where the strain was isolated. The time scale is indicated at the bottom.

Phylogeographic analysis incorporating 9 closely related ST1076 genomes from public databases (BV-BRC database; see Supplementary Table S2 for Genbank BioSample) showed that the 22-strain outbreak clade clustered tightly with ST1076 isolates previously reported from Ningbo, another city in Zhejiang Province (Figure 3 and Supplementary Table S2; Jin et al., 2025; Hu et al., 2021). In contrast, the divergent isolate NY15 grouped with strains of Hangzhou origin, also within the same province.

To estimate the timing of the Deqing ST1076 outbreak lineage, Bayesian time-scaled phylogenetic inference was performed using BactDating. A strong temporal signal was confirmed by the positive correlation between root-to-tip genetic distances and dates of isolation (*R*^2^ = 0.85, *p* < 1.00×10^−4^). Bayesian time-scaled phylogenetic inference was performed with an MCMC chain length of 108 iterations. Convergence was achieved for all estimated parameters (clock rate, MRCA date, and effective population size), with effective sample sizes (ESS) > 200 and visual inspection of trace plots showing good mixing (Supplementary Figure S2). The most recent common ancestor (tMRCA) of the Deqing ST1076 outbreak lineage (excluding the outlier isolates NY15 and NY504) was dated to approximately January 2020 (95% highest posterior density interval: May 2019–May 2020). This estimate suggests cryptic introduction or undetected early circulation of the clone several months prior to the earliest detected isolate (NY14). This pattern of clustering with Ningbo and Hangzhou isolates indicates intra-provincial dissemination of ST1076 within Zhejiang, likely facilitated by patient transfers, shared healthcare networks, or regional referral patterns among hospitals in the province.

## 4 Discussion

CRPA remains a critical priority pathogen on the WHO list (Tacconelli et al., 2018), despite its recent downgrading from “critical” to “high” in the 2024 update, due to its multidrug resistance, high mortality, and propensity for nosocomial transmission. This study provides the first detailed genomic characterization of CRPA in Deqing County, Zhejiang Province, revealing *bla*_KPC–2_ as the predominant carbapenemase (95.5% of CPPA isolates) and ST1076 as the leading clonal lineage (23%).

Carbapenem resistance mechanisms in *P. aeruginosa* include loss of the OprD, efflux pump overexpression, and/or upregulation of chromosomally encoded intrinsic AmpC β-lactamases (Li et al., 2012; Pang et al., 2019); however, horizontal transfer of carbapenemase-encoding genes is recognized as the primary factor (Zhen et al., 2022). According to previous studies (Reyes et al., 2023), the types and prevalence of carbapenemases carried by CRPA strains vary significantly across different regions. Specifically, only 2% of CRPA isolates from the United States carry carbapenemases, whereas 30–69% of isolates from other regions harbor these enzymes: 69% in South and Central America (primarily KPC-2 and VIM-2), 57% in Australia and Singapore (NDM-1 and IMP-1), 32% in China (KPC-2), and 30% in the Middle East (VIM-2 and GES-5). Among these, KPC-2 is the most prevalent carbapenemase globally, followed by VIM, IMP, NDM, and GES (Reyes et al., 2023; Hu et al., 2015). In this study, carbapenemase-producing *Pseudomonas aeruginosa* (CPPA) accounted for 44% (44/100) of the isolates, of which 95.5% (42/44) produced KPC-2. Four CPPA isolates co-carried *bla*_IMP–10_ and *bla*_KPC–2_, a combination that enhances resistance and complicates treatment.

*P. aeruginosa* high-risk clones exhibit high prevalence and diversity of acquired resistance elements, and the transmission patterns of these high-risk strains vary significantly across different regions (Sastre-Femenia et al., 2023). According to previous research, the world-wide top 10 *P. aeruginosa* high-risk clones includes ST235, ST111, ST233, ST244, ST357, ST308, ST175, ST277, ST654, and ST298 (Del Barrio-Tofiño et al., 2020). In a global observational prospective cohort study, ST463- *P. aeruginosa* was the prevalent clone type among CRPA isolates in China (Reyes et al., 2023). However, in this study, MLST analysis revealed high genetic diversity among the 100 CRPA isolates, with a total of 44 distinct STs identified (included a novel ST, ST5216); ST1076 (23%) and ST463 (17%) were the dominant clone types. MLST analysis confirmed the predominance of ST1076-*P. aeruginosa* strains, which further supports the existence of significant regional differences in the clonal types of *P. aeruginosa*. Additionally, previous studies indicate that ST1076 represents a high-risk clone associated with transmission at a hospital in Ningbo, Zhejiang Province (Qiao et al., 2025). This supports regional clonal spread of ST1076 in China, which may be driven by hospital transmission (87% of ST1076 cases involved ICU exposure) and mobile genetic elements. Notably, 87% of ST1076 isolates produced KPC-2, indicating that ST1076-PA may be associated with the spread of KPC-2-mediated carbapenem resistance among CRPA isolates.

While IncU and IncP-6 plasmids are the major *bla*_KPC–2_ carriers in *P. aeruginosa* globally, and in China ST463 strains predominantly carry the conjugative pT1-KPC plasmid with low fitness cost and IS*26*-driven amplification (Letizia et al., 2025; Li et al., 2024), the *bla*_KPC–2_ genes in our collection resided on two non-mobilizable IncU plasmid clusters (AF809 and AE788) that carried no additional resistance genes. This suggests that clonal expansion of ST1076 and ST463, rather than horizontal plasmid transfer, drove local KPC-2 dissemination. The large AF809 cluster (∼390 kb) was exclusive to ST1076, whereas the smaller AE788 cluster (∼33–53 kb) occurred across multiple STs, implying distinct plasmid acquisition histories. Chromosomal *bla*_KPC–2_ in a few ST1816 and ST244 isolates further supports vertical heritability as an alternative persistence mechanism.

Numerous studies have demonstrated that CZA exhibits excellent *in vitro* and clinical efficacy against a large proportion of *P. aeruginosa* strains (Kazmierczak et al., 2018); however, CZA-resistant PA isolates have been increasingly reported worldwide. In *P. aeruginosa*, aside from MBL-producing strains, CZA resistance is primarily mediated by β-lactamase mutations, structural modifications, and efflux pump overexpression (Berrazeg et al., 2015; Kazmierczak et al., 2018). Notably, the predominant ST1076 *P. aeruginosa* clone identified in this study showed high CZA susceptibility (95.7%) (expected, due to avibactam inhibition of KPC-2), in contrast to 40% CZA resistance among ST1076 isolates from Ningbo—where resistance is linked to enhanced biofilm formation and efflux pump activation (Qiao et al., 2025). This discrepancy is likely attributed to lower CZA usage in Deqing, reducing selective pressure for resistance development. In comparison, the ST463 subtype exhibited considerably lower CZA susceptibility (29.4%). Although ST463 isolates also harbored *bla*_KPC–2_ in this study, the reduced susceptibility to CZA suggests the presence of additional resistance mechanisms, such as *bla*_KPC–2_ mutations and efflux pump overexpression that are not inhibited by avibactam (Berrazeg et al., 2015; Kazmierczak et al., 2018). This is consistent with reports of high CZA resistance in Chinese ST463 CRPA strains: a 2021–2022 molecular epidemiology study of two Hangzhou tertiary hospitals found ST463 to be the predominant sequence type among CZA-resistant isolates, accounting for 76.3% of all resistant strains (Hu et al., 2026). Collectively, these findings emphasize that CZA administration should be guided by local antimicrobial susceptibility testing rather than regional or global trends.

## 5 Limitations

This study has several limitations. First, although we state that ST1076 was the predominant clone, this finding reflects data from a single center and is contextualized using published reports from the region, without accounting for CRPA isolates circulating in other hospitals in Deqing City or Zhejiang Province during the same period. Second, the observed predominance of ST1076 appears to be closely linked to the hospital outbreak described in this study. This may limit the generalizability of our findings to other healthcare settings, where different clonal distributions could have been present. Future multicenter studies are needed to further validate these findings.

## 6 Conclusion

This study represents the first epidemiological investigation of CRPA isolates in Deqing County, Zhejiang Province. The results indicate that ST1076 is the predominant strain in the region, primarily carrying the *bla*_KPC–2_ gene, followed by ST463. Genomic analysis indicates that since mid-2020, the clonal spread of ST1076 has been concentrated primarily in ICUs, with intra-provincial transmission linked to strains originating from Ningbo and Hangzhou. Despite high resistance to carbapenems, ST1076 remains highly susceptible to CZA, whereas ST463 is resistant. These findings highlight the emergence of an ST1076 clone associated with KPC-2 transmission in the Zhejiang region, where CZA currently represents a viable treatment option. Therefore, there is an urgent need to strengthen surveillance and infection control measures.

## Data Availability

The datasets presented in this study can be found in online repositories. Genome sequences of all strains have been deposited in the NCBI database under BioProject accession numbers PRJNA1434323.

## Footnotes

1
https://github.com/tseemann/mlst

2
https://github.com/tseemann/abricate

## Supplementary material

Supplementary Figure S1 Drug resistance profile of the 100 CRPA isolates. **(a)** Percentage of MDR, XDR, and non-MDR in carbapenem-resistant *P. aeruginosa*. MDR, multidrug-resistant; XDR, extensively drug-resistant. **(b)** Enzyme-specific distribution of carbapenemase-producing *P. aeruginosa*.

Supplementary Figure S2 Bayesian phylogenetic convergence assessment.

Supplementary Table S1 Characteristics of blaKPC-bearing plasmids reconstructed using MOB-suite.

Supplementary Table S2 The metadata of the 9 closest genomes to our 23 ST1076 CRPA isolates in the BV-BRC database.
