# Supplementary figures and images for: Emergence of ST1076 as a dominant high-risk clone carrying blaKPC–2 in carbapenem-resistant Pseudomonas aeruginosa from Deqing, Zhejiang, China: a 5-year genomic epidemiology study
Source: Front Microbiol. 2026 Jul 9;17:1839819. doi: 10.3389/fmicb.2026.1839819 (PMC13391571; doi:10.3389/fmicb.2026.1839819)

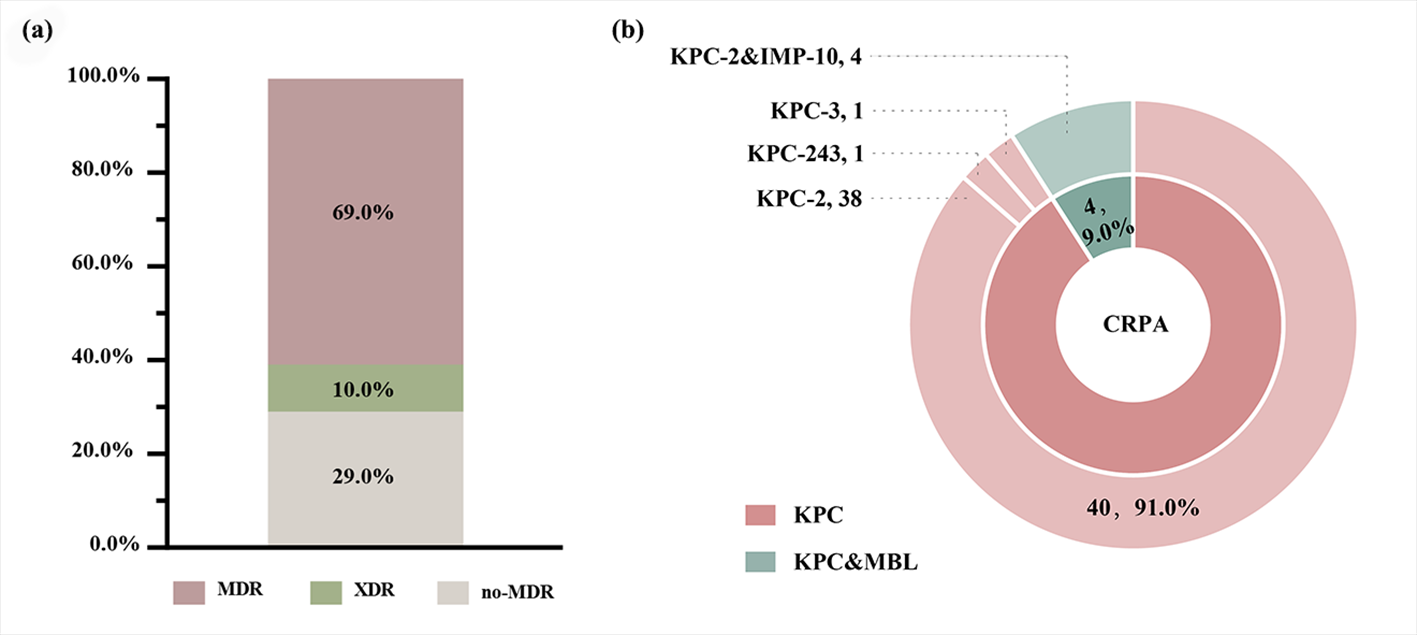

Supplement: Supplementary Figure S1 — Drug resistance profile of the 100 CRPA isolates. (a) Percentage of MDR, XDR, and non-MDR in carbapenem-resistant P. aeruginosa. MDR, multidrug-resistant; XDR, extensively drug-resistant. (b) Enzyme-specific distribution of carbapenemase-producing P. aeruginosa. [file Image_1.tif]

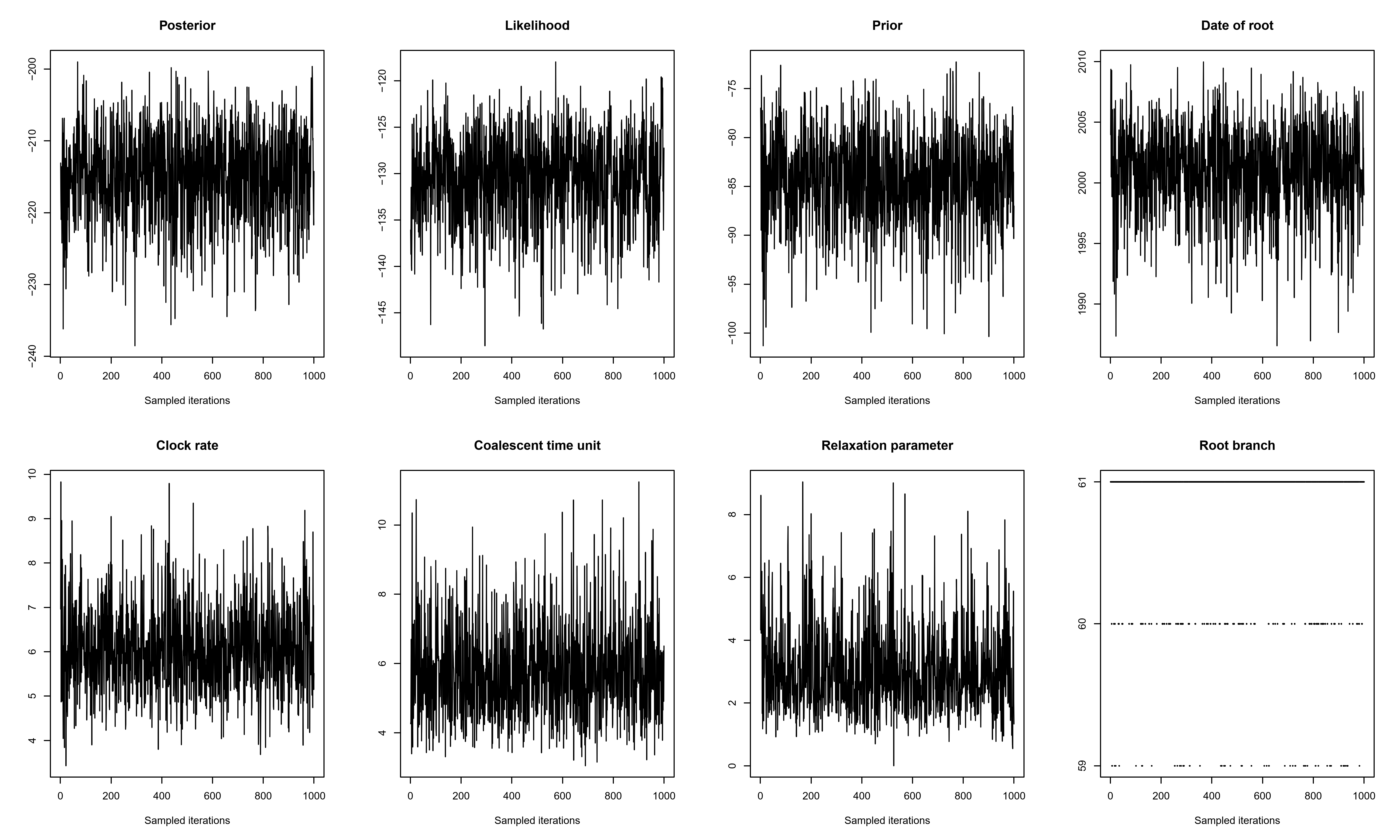

Supplement: Supplementary Figure S2 — Bayesian phylogenetic convergence assessment. [file Image_2.tif]
