# Supplementary material for: Emergence of ST1076 as a dominant high-risk clone carrying blaKPC–2 in carbapenem-resistant Pseudomonas aeruginosa from Deqing, Zhejiang, China: a 5-year genomic epidemiology study
Source: Front Microbiol. 2026 Jul 9;17:1839819. doi: 10.3389/fmicb.2026.1839819 (PMC13391571; doi:10.3389/fmicb.2026.1839819)
Supplement: Supplementary Table S2 — The metadata of the 9 closest genomes to our 23 ST1076 CRPA isolates in the BV-BRC database. [file Table_2.docx]

**Table S2. Detailed information of ST1076 *P. aeruginosa* strains reported in the BV-BRC database.**

| **Sample name** | **Genbank BioSample** | **Collection year** | **Country** | **Location** | **Mash distance** | **Kmer counts** | **P Value** | **Reference** |
| --- | --- | --- | --- | --- | --- | --- | --- | --- |
| S9 | SAMN21988691 | 2016 | China | China: Zhejiang | 0.000167546 | 993/1000 | 0 | [1] |
| S10 | SAMN21988692 | 2016 | China | China: Zhejiang | 0.000191626 | 992/1000 | 0 | [1] |
| S11 | SAMN21988693 | 2016 | China | China: Zhejiang | 0.000215742 | 991/1000 | 0 | [1] |
| LHL-11 | SAMN31266255 | 2021 | China | China: Zhejiang | 0.00028831 | 988/1000 | 0 | [2] |
| LHL-37 | SAMN31266280 | 2021 | China | China: Zhejiang | 0.000312573 | 987/1000 | 0 | [2] |
| LHL-20 | SAMN31266264 | 2021 | China | China: Zhejiang | 0.000312573 | 987/1000 | 0 | [2] |
| LHL-1 | SAMN31266246 | 2021 | China | China: Zhejiang | 0.000312573 | 987/1000 | 0 | [2] |
| ZYPA44 | SAMN20856529 | 2019 | China | China: Zhejiang | 0.00165343 | 934/1000 | 0 | N/A |
| ZYPA17 | SAMN20856502 | 2019 | China | China: Zhejiang | 0.00448671 | 835/1000 | 0 | N/A |

[1] Hu, Y., Qing, Y., Chen, J., Liu, C., Lu, J., Wang, Q., Zhen, S., Zhou, H., Huang, L., and Zhang, R. (2021). Prevalence, risk factors, and molecular epidemiology of intestinal carbapenem-resistant Pseudomonas aeruginosa. *Microbiology Spectrum* 9(3), e0134421.

[2] Jin, L., Ye, H., Xu, H., Shahzadi, A., Pan, X., and Lou, D. (2025). Genomic epidemiology and characterization of difficult-to-treat resistant Pseudomonas aeruginosa isolates co-harboring blaOXA-50 and crpP causing bronchiectasis. *Scientific Reports* 15(1), 12932.
